# Recent evolution of the salivary mucin *MUC7*

**DOI:** 10.1038/srep31791

**Published:** 2016-08-25

**Authors:** Duo Xu, Pavlos Pavlidis, Supaporn Thamadilok, Emilie Redwood, Sara Fox, Ran Blekhman, Stefan Ruhl, Omer Gokcumen

**Affiliations:** 1 Department of Biological Sciences, State University of New York at Buffalo, New York 14260, USA; 2 Institute of Computer Science (ICS), Foundation of Research and Technology–Hellas, Heraklion, Crete, Greece; 3 Department of Oral Biology, School of Dental Medicine, State University of New York at Buffalo, New York 14214, USA; 4 Department of Genetics, Cell Biology, and Development, University of Minnesota, Twin Cities, Minnesota 55455, USA

## Abstract

Genomic structural variants constitute the majority of variable base pairs in primate genomes and affect gene function in multiple ways. While whole gene duplications and deletions are relatively well-studied, the biology of subexonic (*i.e.*, within coding exon sequences), copy number variation remains elusive. The salivary *MUC7* gene provides an opportunity for studying such variation, as it harbors copy number variable subexonic repeat sequences that encode for densely O-glycosylated domains (PTS-repeats) with microbe-binding properties. To understand the evolution of this gene, we analyzed mammalian and primate genomes within a comparative framework. Our analyses revealed that (i) *MUC7* has emerged in the placental mammal ancestor and rapidly gained multiple sites for O-glycosylation; (ii) MUC7 has retained its extracellular activity in saliva in placental mammals; (iii) the anti-fungal domain of the protein was remodified under positive selection in the primate lineage; and (iv) *MUC7* PTS-repeats have evolved recurrently and under adaptive constraints. Our results establish *MUC7* as a major player in salivary adaptation, likely as a response to diverse pathogenic exposure in primates. On a broader scale, our study highlights variable subexonic repeats as a primary source for modular evolutionary innovation that lead to rapid functional adaptation.

Duplications, deletions, inversions and translocations of genetic segments, collectively called genomic structural variants, constitute an evolutionarily important form of genetic variants^1^. When two genomes from the same primate species are compared with each other, structural variants constitute 2 to 7 times more base pair differences than single nucleotide variants^2^. A functionally relevant, but understudied subset of structural variants, is copy number variation of tandem repeats within individual coding exons (subexonic repeat variation)^3^. A recent study has shown that, despite the high intrinsic mutability of subexonic repeats, the vast majority of thousands of subexonic repeats remain strongly conserved among mammals^4^. However, there is no study to our knowledge that specifically investigated the evolution of copy number variable subexonic repeats from their initial emergence as functional units to the adaptive constraints and mutational properties that contributed to their extant variation in primates.

Here, we focus on the salivary mucin-7 gene (*MUC7*) to study the evolution and functional impact of subexonic repeats. *MUC7* comprises a number of interesting properties that could make it a model for studying subexonic repeats (Fig. 1A). First, *MUC7* carries subexonic repeats that are 69 bp (23 amino acids) long and are copy number variable in humans^5^. These repeats can be particularly well studied in this gene because the repeats are relatively short and few in number compared to other mucins. Second, the subexonic repeats of *MUC7* code for densely O*-*glycosylated proline-, threonine-, and serine-rich (PTS) tandem repeat domains. PTS-repeats are a hallmark of the mucin group of proteins^6^. Mucins are highly glycosylated proteins that serve a variety of roles in organisms from lubrication to cell-signaling to defense^7^. In addition, the dense arrays of O-glycans also create large protease-resistant regions that provide binding sites for commensal microbes and pathogens, thus preventing them from binding epithelial cells^8^,^9^. Third, *MUC7* codes for an abundant salivary protein^10^. Recent studies have reviewed the importance of structural variants affecting salivary proteins as a response to rapid changes in human diet and composition of oral exposure to different pathogens^1^,^11^. *MUC7* provides an opportunity to assess the broader implications of subexonic repeat variation within the context of the functional evolution of the mucin functional group and primate salivary adaptation.

*MUC7* belongs to the secretory calcium-binding phosphoprotein (SCPP) family, which likely evolved after the divergence of bony and cartilaginous fish^12^. The SCPP gene family resides at the short arm of chromosome 4 in humans. It also includes other salivary proteins, such as histatins, proline-rich proteins, and statherin, as well as non-salivary proteins, such as milk caseins and enamel matrix proteins. In addition to the PTS-repeats, the MUC7 protein contains a non-glycosylated “naked” region at the N-terminus of the apomucin molecule that shares homology with salivary histatins. Similar to them, this region exhibits bactericidal and fungicidal properties^13^. MUC7 also contains two moderately N- and O-glycosylated domains flanking the PTS repeat domain, and a C-terminal domain that contains a leucine-zipper motif^14^. Functionally, the comparatively small non-gel-forming MUC7 contributes relatively more to the innate immune functions of saliva than to the lubricating or barrier-forming properties typical for the much larger gel-forming salivary mucins, such as MUC5B and MUC19^15^,^16^.

Even though MUC7 is grouped with other mucin proteins by virtue of their biochemical and functional similarities, it shares no sequence similarity or known recent common evolutionary ancestor with any of the other known mucin genes^6^. Other than its syntenic relationship with the SCPP gene family and the functional similarity to other mucins, the evolution of *MUC7* remains unclear. Several attempts were made to pinpoint the origins of different mucins and follow their evolutionary trajectories^6^,^17^,^18^,^19^,^20^. The defining feature of mucins, PTS-repeats, have been reported in *MUC7* to range from 5 to 6 haploid copies in humans^21^. Functionally, the less-common 5-copy allele was suggested to be associated with protection against asthma^5^,^22^. From an evolutionary standpoint, the copy number variation of *MUC7* PTS repeats in nonhuman primates have not been fully explored. Therefore, in this study, we conducted genomic analyses to elucidate the evolution of *MUC7* in primates, especially with regards to its subexonic repeats.

## Results

### *MUC7* likely originated in the placental mammal ancestor as a functional gene

**Figure 1:**
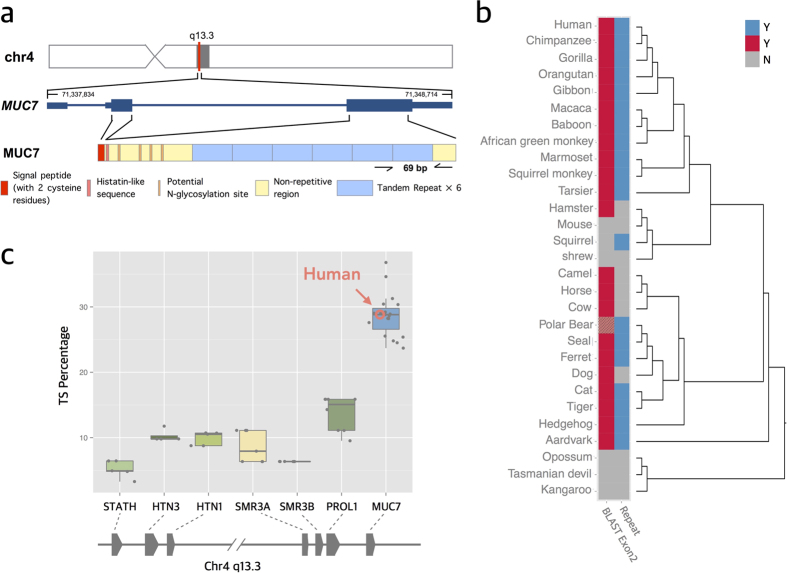
The structure and function of *MUC7.* (**a)** Genomic location, organization and subexonic copy number variation of the *MUC7* gene in the human reference genome (GRCh37/hg19). The gray bar indicates the chromosomal band (q13.3) where *MUC7* resides. The zoomed-in version of the gene shows the two coding exons (thick blue bars), the UTR region at the 3′ and 5′ ends (thin blue bars) and intronic regions (blue line in-between exons). Start and end of the transcript are indicated by the numbers at each end. The different functional domains within the MUC7 protein are designated by different colors at the bottom of the panel^66^; (**b)** Presence or absence of PTS-repeats and of human exon 2 (*i.e.*, first coding exon coding for the conserved signal peptide with cysteine residues detectable by BLAST search). The presence of significantly similar sequences (E-value < 0.01) to human exon 2 is indicated with red and the absence with gray. The polar bear *MUC7* ortholog contains a truncated version of exon 2 with only 36 of 54 bp represented (shown in hatched pattern). The presence of PTS repeats is shown in blue. The absence is shown in grey. The phylogenetic tree on the right indicates the species relationships between the organisms analyzed here; (**c)** Percentage of T and S amino acids (indicative of potential *O-*glycosylation sites) in relation to the total number of amino acids for *MUC7* orthologs and other proteins belonging to the SCPP gene family across mammals. *MUC7* shows a significantly higher TS percentage compared to other salivary proteins in *SCPP* gene family (based on the NCBI gene model; P-value between *MUC7* and *PROL1* is 1.123 × 10^−09^ by one-tailed student’s *t*-test).

*MUC7* is evolutionarily related to SCPP gene family through gene duplication^23^. The evidences for this are the syntenic location of *MUC7* within the SCPP gene cluster and the presence of a signal peptide, which is a functionally fundamental hallmark of all extracellular matrix proteins^24^. However, it is not clear when *MUC7* emerged as a functional gene. Based on current gene prediction models, it appears that *MUC7* orthologs are restricted to placental mammals^25^. However, gene predictions, especially for genes that have repeat content as in *MUC7,* may be error prone^26^. To independently investigate the presence or absence of *MUC7* orthologs, we used BLAST^27^ to identify similar sequences to the non-copy number variable first coding exon (second exon, which codes for the signal peptide) of human *MUC7* in multiple vertebrate reference genomes (Figure S1). Our results are concordant with the gene models where *MUC7* is present in most placental mammals, but absent in marsupials (Fig. 1B). Although *MUC7* orthologs could not be found in rat and mouse reference genomes, it was recently reported that rodents express a salivary mucin gene that functionally resembles MUC7^15^. It is therefore possible that this gene has evolved convergently, filling the adaptive niche for a salivary-specific small mucin in rodents after lineage specific deletion of *MUC7*. There are some caveats in our analyses. First, It is plausible that lineages such as marsupials, where we could not detect *MUC7-*like sequences, originally carried the *MUC7* gene but may have lost it during their evolution, similar to rodents. Second, it is also plausible that the signal peptide sequence, that we used for BLAST analysis, may have diverged in some lineages. Thus, starting from the primate *MUC7* signal peptide sequence, we may not have had the statistical power to detect such diverged sequences in some species. In fact, when we repeated the BLAST analyses using the *MUC7* histatin-like sequence instead, we could detect significantly similar sequences only in primates, even though syntenic histatin-like sequences are present in most mammalian *MUC7* genes (Figure S1). This exemplifies the shortcomings of BLAST analysis for definitively pinpointing to the origin of the *MUC7* gene. Having stated the caveats, still the most parsimonious explanation, based on the analyses presented here, is that *MUC7* evolved in the placental mammal ancestor.

### The ancestral *MUC7* gene has rapidly gained PTS repeats after its emergence

One question is when did *MUC7* gain heavy O-glycosylation through its PTS-repeats. The clusters of T and S amino acids in proteins were discussed in multiple previous studies as targets for O-glycosylation^14^,^28^. In fact, mucins, as a functional group, are defined by the presence of PTS-rich repeats^6^, where T and S clusters provide targets for O-glycosylation. For human MUC7, the heavy glycosylation of the PTS-repeats has been demonstrated experimentally^29^. Therefore, we first asked whether PTS-rich repeats exist in all *MUC7* non-human orthologs. To address this question, we conducted multiple overlapping analyses to minimize biases due to the variation in the quality, completeness and annotation of reference genomes. First, we used BLAST to identify sequences across available mammalian genomes that are similar to the PTS-repeats observed in humans (E-value < 0.01). Second, we investigated the presence or absence of subexonic repeats across mammalian reference genomes as annotated by Simple Tandem Repeat track^30^ as well as through manual inspection within the predicted *MUC7* coding sequences. Collectively, our analysis showed that *MUC7* coding sequences in most placental mammals comprise repeated PTS-sequences (Fig. 1B).

Certain mammalian species, such as dog, cow, horse, camel and shrew also showed no evidence for PTS-repeat sequences. This may either indicate lineage-specific loss of repeats, polymorphism within species, or an artifact resulting from reference genome assembly errors^31^,^32^. It should be noted here that most other mucins, including *MUC1, MUC4,* and *MUC6,* have intact PTS-repeats in mouse^33^,^34^,^35^, although the sequences of these repeats can vary between humans and mice^33^. As such, a complete loss of all PTS-repeats of a mucin gene is probably a rare event in evolution. Thus, the observed lack of *MUC7* PTS-repeats in at least some of the mammalian species mentioned above could well be due to reference genome errors. Indeed, similar incorrect representations of tandem duplications in reference genomes have been reported earlier^36^.

To evaluate whether the PTS-repeats in other mammalian species are defined, as in humans, with high proportion of T and S amino acids, we measured the number of codons encoding for T and S amino acids in the coding sequences of *MUC7* genes in extant mammals (Fig. 1C). We found that, regardless of the number of repeats found in the gene, all *MUC7*-coding sequences in mammals have a proportionally higher T and S content than other members of the evolutionarily related *SCPP* gene family (*e.g., MUC7* T and S percentage is significantly higher than the adjacent *PROL1* among mammals, P-value = 1.123 × 10^−9^, one-tailed Student’s *t*-test). We also compared the proportion of T and S amino acids between *MUC7* and another salivary mucin gene, *MUC5B.* We found that the proportion of T and S amino acids in *MUC7* is also consistently higher than *MUC5B* among mammals (Figure S2, P-value = 0.004382, one-tailed Student’s *t*-test). These results suggest that the ancestral *MUC7* likely gained TS rich, PTS-repeats early after its emergence.

### The chromosomal location of *MUC7* likely determined saliva-specific expression

**Figure 2:**
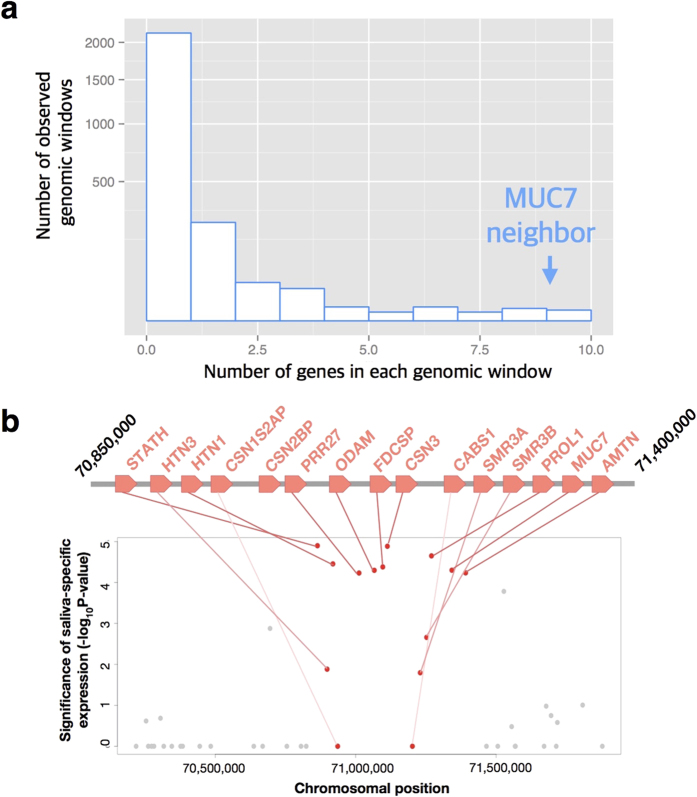
The *MUC7* gene is located within a chromosomal region where most of the genes show saliva-specific expression. (**a)** We searched chromosome 4 for 15-gene windows (*i.e.,* each window is defined by 15 adjacent genes). Then, for each gene we calculated the likelihood of saliva-specific expression by comparing the expression of this gene in minor salivary glands as compared to all other non-salivary tissues in the GTEx dataset using a Wilcoxon rank-sum test (See methods for the details). The x-axis shows the number of genes in a given window that are expressed primarily in saliva (*i.e.* expression in minor salivary gland is significantly higher than in other tissues (P-value < 0.01)). *MUC7* is located in the window with the highest number of genes having salivary gland-specific expression (10 genes). The y-axis shows the frequency of observed windows in squared-scale (Wilcoxon rank-sum test, P-value = 2.2 × 10^−16^); (**b)** This plot shows the likelihood of saliva-specific expression for the chromosomal region that *MUC7* resides on chromosome 4. In the scatter plot, the y-axis is the P-value (same way the P-value is calculated for the histogram in Fig. 2A) for saliva-specific expression. The x-axis is the chromosomal location on chromosome 4. The gray dots indicate the genes flanking the “window” in which *MUC7* resides, while red dots indicate genes in the same window. The cartoon above the scatter plot is a zoomed-in depiction of the location of *MUC7* and the SCPP cluster of genes that show high saliva expression.

*MUC7* is expressed primarily in saliva, and several other genes in the *SCPP* gene family are also expressed in saliva^12^. Once a gene duplication happens, such as the one which led to initial genesis of *MUC7,* it is plausible that the cis-regulatory sequences of the ancestral gene are also copied, thus regulating the expression of the duplicated gene. Indeed, recent studies have shown that tandemly duplicated genes have an increased chance of having similar expression profiles^2^,^37^. To test whether the saliva-specificity of *MUC7* expression is similar to the neighboring members of the *SCPP* family, we used a global gene-expression database, GTEx^38^ (Table S1). Briefly, we compared the expression in minor salivary gland (the only salivary gland presented in GTEx dataset) to the expression in all other GTEx tissues. Then, we calculated a nominal P-value for saliva-specific expression for all genes across the genome. This analysis showed there is an unexpectedly high number of genes with saliva-specific expression adjacent to *MUC7* when compared to the rest of the genome (Wilcoxon rank-sum test, nominal P-value = 2.2 × 10^−16^, Fig. 2A,B). Thus, it is plausible that the gene cluster where *MUC7* emerged has had saliva specific expression for a long evolutionary time and that the location of its emergence predetermined the expression pattern of of *MUC7.*

### The *MUC7 PTS-repeats* exhibit very high and recurrent copy number variation in mammals and primates, but remain T and S rich

To better understand the evolution of PTS-repeat copy number, we tested whether the PTS-repeat copy number between-species variation is unusual as compared to other subexonic repeats across the genome. To do this, we used a recently available database^4^, which characterized subexonic repeat content in dozens of different eukaryotic genomes. According to their analysis, *MUC7* PTS-repeats were in the upper 0.4% and 17% for subexonic repeat cross-species divergence in nucleotide and copy number divergence, respectively (Figure S3).

**Figure 3:**
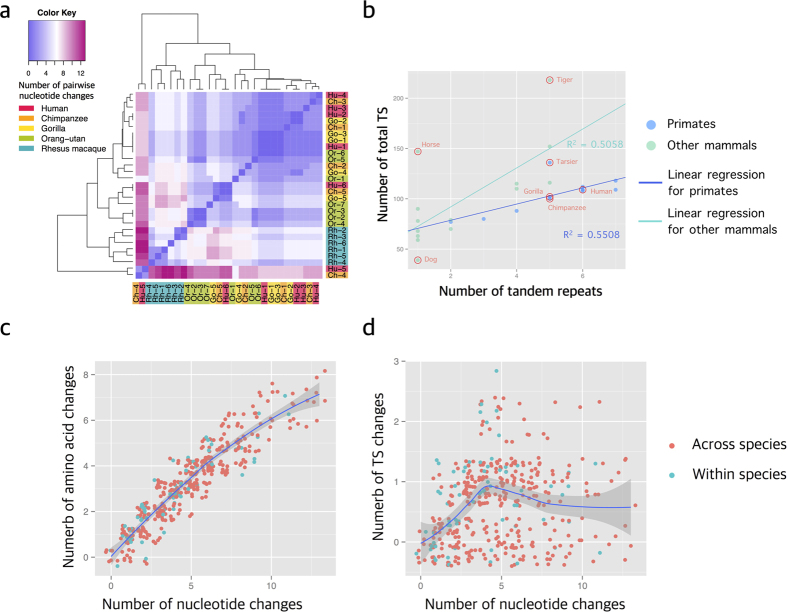
Presence of *MUC7* gene and PTS tandem repeats in mammals. (**a)** Heatmap of pairwise nucleotide differences between each repeat sequence within and among primate *MUC7* PTS-repeats. Different colors on the right and bottom axes indicate different species. The number shows the position of those repeats in their *MUC7* repetitive regions (*e.g.,* Rh_1 indicates the first repeat from the 5′ in the rhesus macaque reference genome). The colors in the heatmap show the nucleotide differences between each pair of repeats, with warmer colors indicating a higher number of nucleotide differences. The groupings (clusters) shown on top and to the left of the heatmap were constructed based on sequence similarity without any *a priori* hypothesis. Note that, if there is no recurrence and most repeats share a common ancestor, then we expect to see clustering of orthologous repeats. Instead, we observed clustering of repeats within species, indicating species-specific duplication events; (**b)** Number of total T and S amino acids in each *MUC7* protein in relation to the number of TS tandem repeats in primates and other mammals. Relevant species and apparent outliers were indicated by a red circle and their names on the graph. If a species did not show any subexonic repeat content, it is designated by 1 on the x-axis; (**c)** For each repeat in primate species, the number of pairwise amino acid changes is strongly correlated with pairwise nucleotide changes both across species and within species (R^2^ = 0.8643), while (**d)** the number of pairwise TS amino acid changes are not (R^2^ = 0.03266). The numbers of pairwise TS amino acid remain similar within and among primate species.

To further understand the evolution of the between-species divergence, we tested whether recurrent evolution of PTS-repeats explains some of the observation among primates. To do this, we constructed a heatmap based on the pairwise difference matrix between individual repeat sequences available in reference genomes among primate species (Fig. 3A). We observed that all of the repeat sequences in rhesus macaques cluster with each other, indicating a burst of independent duplication events in the rhesus macaque lineage. Similarly, 3 out of 7 repeat sequences in the orangutan cluster with each other, indicating an expansion of these repeats within the orangutan lineage. We also confirmed these observations using a strongly supported phylogenetic tree based on individual repeat sequences in great apes (Figure S4), as well as using only synonymous SNPs (Figure S5) to ensure that our observations are not due to functional convergence. PTS-repeats in the analyzed species remain in-frame, 69 coding nucleotides in all the species observed, reducing the potential alignment errors. Overall, our results suggest that some of the individual repeats evolved recurrently through lineage-specific duplication events in different primate species.

To investigate the effect of PTS-repeat copy number on potential O*-*glycosylation sites within the MUC7 protein, we plotted the number of repeats in mammalian species over the proportion of T and S amino acids in these proteins (Fig. 3B, Table S2). We found them to be highly correlated for both non-primate mammals (R^2^ = 0.5058) and primates (R^2^ = 0.5508). If TS-dependent O-glycosylation of these repeats does not have a major fitness effect (*e.g.*, under neutral evolution), mutations should accumulate and revert to a lower percentage of clustering of T and S content than expected by chance. Indeed, the number of nonsynonymous differences from the pairwise comparison of individual repeats within and between species strongly correlate with the number of synonymous differences (R^2^ = 0.8643, Fig. 3C). However, for T and S amino acids the numbers remain similar, independent of overall nucleotide differences of individual repeats from each other (R^2^ = 0.03266, Fig. 3D). This supports the notion that TS-dependent O-glycosylation is the main feature that is under adaptive constraints.

In addition to the above-described analysis, we considered that the enzymes, namely the GalNAc transferases, confer O-glycosylation to the polypeptide backbone in a site-specific manner^39^. This implies that in addition to the number of T and S amino acids, their relative positioning and adjacent amino-acids determine the overall glycosylation potential of the protein. Specifically for *MUC7,* GalNAc transferases are known to target PTS sequences in such a site specific manner^39^. To test to what extent these recognition motifs are conserved, we aligned all of the primate mucin repeats to each other. This analysis revealed that the GalNAc transferase recognition motifs as they were defined in humans^39^, are clearly retained among primates (Figure S6). Overall, our findings show that both the number of T and S amino acids as well as their context within individual PTS repeat domains remain highly conserved. This points to O-glycosylation as the primary driver of adaptive evolution within MUC7 PTS repeats.

### Copy number variation of *MUC7* PTS repeats has likely evolved under adaptive constraints

**Figure 4:**
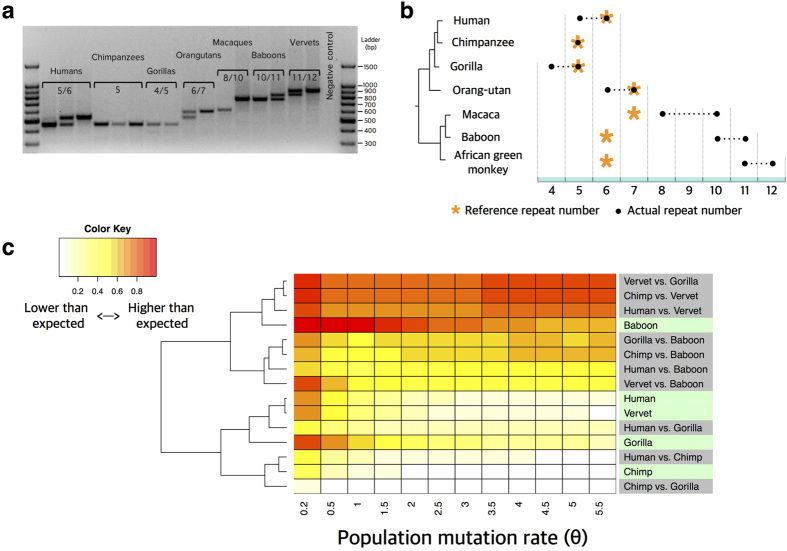
Copy number variation of *MUC7* PTS-repeats in primates. (**a**) Copy number variation of *MUC7* PTS-repeats genotyped by PCR (Table S3); and (**b**) compared to the reference genome copy number as indicated by asterisks. The black dots and dashed lines indicate the range of observed PTS repeat copy numbers; (**c)** Heatmap depicting the comparison of simulated (Table S4) and observed (Table S3) PTS-repeat copy number variation. The x-axis shows the mutation rates that we used for simulations (*e.g.,* θ = 1 corresponds to 1 duplication/deletion event per million years). For each cell in the heatmap, we calculated the ratio between the expected and observed copy number variation. The red colors indicate higher observed variation as compared to simulated variation, whereas the white colors indicate the opposite. The dendrogram to the left is constructed without any *a priori* input by hierarchical clustering of the values in individual cells in each row. The row labels on the right indicate within species comparisons (green) and among species comparisons (gray). There is consistently more-than-expected variation among great apes and old-world monkey species (upper rows) than within-species and within-families (lower rows). The within-species variation in baboon does not fit in this general trend, possibly because of ascertainment bias due to small sampling.

To further understand the adaptive forces that have shaped *MUC7* PTS-repeat copy number variation in primates, we documented copy number variation of *MUC7* PTS-repeats within and across primates, using polymerase chain reaction (PCR)-based genotyping across 10 non-human primate species (Fig. 4A, Table S3). Our results revealed *within-species* copy number variation for the PTS-repeats (Fig. 4B) where 3 out of 4 great ape species showed common subexonic copy number variation in this gene. We extended our analysis to Old World monkeys, and despite the very limited number of samples we surveyed, we observed copy number variation of PTS repeats. Note that the Old World monkey reference genomes consistently harbor lower numbers of mucin PTS-repeats than we observed by PCR-based assays in non-human primates. This may be due to either uncaptured variation in our sample or misassembly in the reference genomes. Regardless, *MUC7* PTS-repeats show high levels of *intra-species* copy-number variability in the majority of primate species. In general, within-species exonic copy number variation is rare and recurrent, within-species variation in multiple species is even rarer. Such variants have often associated with adaptive, and on occasion, diversifying selection^40^,^41^,^42^,^43^,^44^. To sum these results, it is clear that the mutation rate (*i.e.*, the rate of gain and loss mutations) is high for *MUC7* PTS-repeats, contributing to within-species copy number variation. However, the range of copy number variation (*i.e.*, 4 to 7 in great apes) is relatively low considering the high mutation rate and may have been shaped with species-specific adaptation. Therefore, we hypothesized that the copy number of PTS-repeats evolves under a fast mutation rate, but was confined to an adaptive range in each individual species.

To test our hypothesis, we simulated the expected range of copy number within and across great-ape species under neutral expectations and under various mutation rates of copy gain/loss and different ancestral copy-number states (Table S4). Since we saw recurrent copy number gain and loss independently within humans, gorillas and orangutans, we surmised that at least one gain or loss has happened in each of these lineages. The average coalescence time for these species are not more than 500,000 years ago and, for humans much less^45^. Since we observed copy number variation within orangutans, gorillas and humans independently, we can assume a conservative mutation rate of at least one copy number gain or loss every 500,000 years. Therefore, we considered mutation rate scenarios ranging from 0.5 to 2 copy-number-changes every million years. Our results have shown that the observed cross-species variation among great-apes is significantly lower than expected when we extrapolate the mutation rate from within species variation (Wilcoxon rank-sum test, P-value < 0.01, Figure S7).

These simulations are simplistic as they do not take into account the effective population size of each species. Thus, we simulated genetic variation under a Wright-Fisher model of neutral evolution, taking into account both speciation and the process of coalescence. We used a combination of ms-based tools^46^ and the newly developed algorithm CoMuS (see methods for details) to obtain polymorphic tables for copy number variation data. We assumed a conservative range of theta (*θ*) values from 0.2 to 5.5 (*i.e.*, corresponding to a range of mutation rates from 10^−6^–10^−4^ mutations per locus per generation, assuming N_e_ = 10,000) (Table S4). To take into account potential negative fitness effects due to extremely high PTS copy numbers, we incorporated a strong selection coefficient for copy numbers higher than 12. We chose this upper limit as it is the highest copy number we observed among primates. It is important to note that the inclusion or exclusion of this upper limit in our simulations does not change the trends we observed in this analysis. Using this approach, we generated the null distributions of copy number variation within and across species under neutrality. We then compared the neutral distribution of copy-number variation to the observed copy-number variation within and across-species variation for species where we have genotyped more than 3 individuals (*i.e.*, 6 haplotypes) (Table S3). We found that the observed copy number variations within species (except for baboons) and among great apes are lower than the simulated variations (Fig. 4C). However between great apes and old world monkeys (vervets and baboons), we observed more-than-expected copy number variation as compared to simulated data. The increase in variation within baboons in Fig. 4C is due to the fact that all three individuals that we genotyped were heterozygous. It is plausible that further sampling would help to resolve this issue more conclusively. Overall, our results show that expansions or reductions of the copy number of *MUC7* PTS-repeats occur mostly between different primate species. In contrast, within-species variation remains relatively stable.

### Different adaptive pressures have likely shaped the extant genetic variation of *MUC7* functional domains

**Figure 5:**
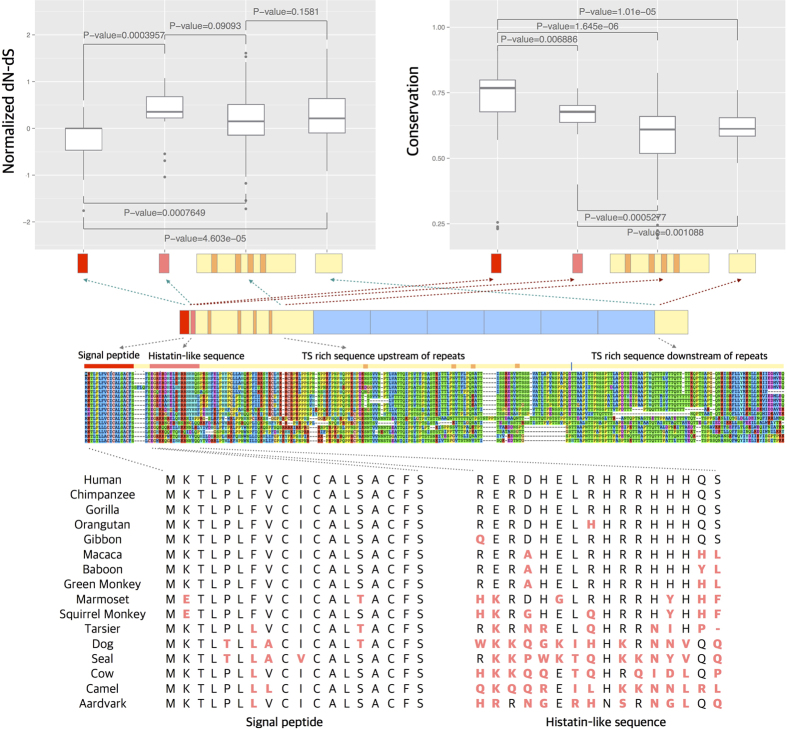
The adaptive evolution of *MUC7* in mammals. The upper-left panel shows the normalized ratio of nonsynonymous to synonymous variation for each functional segment of *MUC7*. The upper-right panel shows the overall nucleotide-level conservation for the same functional segments. The protein alignments of the signal peptide and the histatin-like domain that underlie these graphs are shown at the bottom. We did not include the PTS-repeat region in this analysis because copy number changes and sequence similarity introduce major alignment problems. P-values were calculated using a one-tailed Wilcoxon rank-sum test.

To understand the adaptive forces shaping the non-repeated regions of the *MUC7,* we tested the presence of positive selection using the PAML package^47^ for each of the well-supported branches in the phylogenetic tree using *MUC7* coding sequences outside of PTS-repeats across different mammalian clades (Figure S8). Our results suggest the action of positive selection on the branch leading to the primate ancestor for the *MUC7* gene (PAML-codeml, P-value = 3.771 × 10^−5^, see Table S5 for the detailed results of the PAML analysis). The signal peptide of MUC7, which is important for transport of the protein outside of the cell^14^, remains highly conserved across mammals (Fig. 5), indicating negative selection. It is important to note that signal peptide is post-translationally cleaved from the mature MUC7 protein. In contrast, the histatin-like sequence of the gene, which is involved in antifungal activity^48^, shows a significant change in amino-acid sequences in primates as compared to non-primate mammals (Fig. 5). Indeed, when we individually tested these regions with PAML for positive selection, the histatin-like sequence, but not the sequence that codes for the *signal peptide* of the gene, showed evidence for positive selection (P-value = 0.002) in the primate lineage (Table S5). Nevertheless, the non-repeated N- and O-glycosylated sections of the gene, which include many alignment-gaps, as well as a high level of sequence diversity, retain the general “bottle brush-like” structure^14^. Briefly, the bottle brush-like structure refers to a high proportion of T and S amino-acids, which are potential targets for O-glycosylation, punctuated with P amino acids, which are important for maintaining the rigidity of the protein. This layout would then lead to a rigid linear structure with multiple O-glycans attached to this structure. We also observed relatively high level of change in the 3′-end of the gene, which encodes for a leucine-zipper motif in humans^14^. The domain may contribute to the overall stability of the highly glycosylated structure of MUC7 protein^49^ while the PTS-repeats determine the overall O-glycosylation of the protein. The leucine-rich domains of the protein seem to have evolved in the Simian lineage after the divergence of tarsiers (Fig. 5), and as such may have attained a relatively recent functional relevance in primates.

## Discussion

We documented extensive genetic variation in *MUC7* that has been shaped by multiple, species-specific adaptive constraints. Concordant with its role as an innate immunity component, MUC7 was reported to interact with a multitude of oral microorganisms and extra-oral pathogens^50^,^51^,^52^. Therefore, the increased *MUC7* genetic diversity in primates could be a consequence of diverse pathogenic pressures, as suggested by the Red Queen hypothesis^53^.

Our results draw an initial picture of the evolution of *MUC7* at three levels corresponding to three functional roles of the protein. At the first level, the signal peptide has evolved under negative selection, maintaining the extracellular activity of the protein. At the second level, the histatin-like domain has evolved under directional selection in the primate lineage, possibly to counteract a particular fungal pressure. At the third level, the copy number of PTS-repeats has evolved under lineage-specific adaptive constraints, likely determining the O-glycosylation repertoire of the protein, which is important for rheological and microbial binding properties of *MUC7*^52^,^54^.

*MUC7* provides a glimpse into adaptation through recurrent evolution of subexonic repeats. Copy numbers of most subexonic repeats normally remain highly conserved among mammals^4^, implying the strong influence of negative selection. The few subexonic repeats that remain copy number variable within species provide a distinct adaptive fodder for rapid evolution as hypothesized elsewhere^55^,^56^. First, the copy number of these exonic repeats changes rapidly^57^. Second, copy number variations of these sequences affect larger portions of the proteins than single nucleotide variations. Therefore, directional positive selection can lead to rapid increase or contraction of subexonic repeats in a species-specific manner. Examples of such rapid evolution of copy number variation of subexonic repeats in humans were demonstrated for the dopamine receptor gene^58^, as well as subexonic DUF1220 domain in multiple genes^59^. For *MUC7*, it is likely that the number of subexonic PTS-repeats, each carrying potential O-glycosylation sites, adaptively fine-tune the amount of O-glycosylation of the entire mucin protein in individual primate lineages. Indeed, similar mechanisms may play important roles in the evolution of other mucins. They may also affect the copy number variable cysteine-rich domains (CYS), which are functionally important in some mucin genes but apparently absent in *MUC7*^31^. Overall, we anticipate that rapidly and recurrently evolving subexonic repeats similar to the ones of *MUC7* will be discovered in other primate genes as targets of species-specific adaptation.

## Methods

DNA Samples from non-human primate samples were purchased from Coriell Institute. The non-human primate samples that were genotyped can be found in Table S3. We used PCR to amplify the region that contains the entire PTS-repeat region of *MUC7* in both human and other primates. The details for both experimental, bioinformatic and population genetic analysis can be found in below. We provide the R codes and sequence alignments that are used in this study in our website (gokcumenlab.org).

### PCR Genotyping

PCR were performed in a total volume of 25 μl per reaction with 2.5 μl of 10 × standard *Taq* Reaction Buffer (10 mM Tris-HCl, 50 mM KCl, 1.5 mM MgCl_2_), 0.5 μl of dNTP (10 mM), 1 μl of MgCl_2_ (25 mM), 1 μl of each primer, 0.625 units of *Taq* DNA Polymerase (BioLabs Inc.), and 20–40 ng DNA template. The primers are listed in Table S2.

### Bioinformatic and evolutionary analysis

To determine the presence of subexonic repeats across mammalian MUC7 orthologs, we used (i) BLAST (E-value < 0.01) to identify sequences that are similar to *MUC7* human PTS-repeats and Simple Tandem Repeat track^30^, as well as through manual inspection. The genomic location information of *MUC7* is on chr4: 71337834-71348714 (GRCh37/hg19) as defined by RefSeq gene track (NM_152291). Coding sequences of *MUC7* in other mammals were extracted from GenBank^60^. The alignments were conducted both by Clustal Omega^61^ and Muscle^62^, followed by manual curation to reduce alignment errors. The conservation of MUC7 amino acid sequence was calculated by using Jensen-Shannon Divergence-based method^63^. The T and S amino acid content for *MUC7* among different species was calculated by MEGA 6.0 after alignment^64^. A maximum-likelihood tree was built with *MUC7* sequences across mammals using MEGA 6.0^64^ under JTT with freq. (+F) model, and gamma distributed with invariant sites (G+I) with 500 bootstrap replicates (Figure S7).

### Copy number simulations

We simulated the copy number changes for Great apes (Human, Chimpanzee, Gorilla and Orangutan) under different copy number gain-loss rate (0.5/1.0/1.5/2.0 copies per million year). Based on the species phylogeny of these four great ape species, we assumed that the original copy number state for the common ancestor of Great apes was 5 copies 11 million years ago. Then every 1 million year, there is a random gain or loss of 0.5/1.0/1.5/2.0 copies for Orangutan and the common ancestor of Human, Chimpanzee and Gorilla separately. At 8 million years ago, the common ancestor of Human and Chimpanzee separated from Gorilla and they started the copy number gain and loss simulation separately. The same simulation continues to 5 million years ago that Human and Chimpanzee separated from each other, and start their copy number gain/loss process independently until present. We simulated this process 1,000 times for 4 different copy-number-change rates (0.5/1.0/1.5/2.0 copies per million year), and for each simulation, calculated the variation of final state of simulated copy numbers for Human, Chimpanzee, Gorilla and Orangutan. The observed copy number state in present is Human 5 or 6 copies, Chimpanzee 5 copies, Gorilla 4 or 5 copies, and Orangutan 6 or 7 copies. The variations of observed copy number state in present are significantly less than variations simulated under neutrality (wilcoxon rank-sum test, P-value < 0.01).

We used CoMus (Coalescent of Multiple Species, http://pop-gen.eu/wordpress/software/comus-coalescent-of-multiple-species) to obtain within- and between-species polymorphisms. Assuming that *k* mutational events (gain or loss of copies) occur per million years, θ value is calculated by 4 × 25 × 10^4^ × *k*/10^6^ = *k* (effective population size for humans 10^4^, and the generation time is set to 25 years). In our simulations, *k* varied between 0.2 to 5.5 mutational events per million years. Furthermore, we obtained a phylogenetic tree for Human, Chimpanzee, and Gorilla from the UCSC website (http://www.ucsc.edu/) and used it as a reference tree for the simulations. We assumed that the effective population size (N_e_) is constant for each primate species and that N_e_ for Chimpanzees is twice the human N_e_. Given a reference tree, and population parameters, CoMuS generates polymorphic samples similar to Hudson’s *ms*^46^. Then, we used microsat.c code provided in the *ms* software package to convert polymorphic samples to copy-number polymorphisms. Simulated datasets were used to obtain the null distribution of copy-number variation. Then, P-values were calculated by comparing the observed copy-number variability to the simulation-generated null distribution. This calculation was performed with the supposition that a copy number above 12 PTS-repeats is maladaptive, given that this is the highest copy number observed among primates. It is important to note that our results regarding the adaptive constraints on the PTS-repeats are partially dependent on the mutation rate of subexonic repeats, which have been shown to vary widely^4^,^65^.

### Analysis for gene with salivary-specific expression

The dataset from gene-expression database, GTEx^38^ was used for the expression analysis (GTEx Analysis V4/RNA seq/Gene RPKM). We compared the expression in minor salivary gland to the expression in all other glands (n = 52) for all the genes in the human genome. On chromosome 4, Wilcoxon rank-sum test was applied to find the genes with significantly higher than expected expression (upper 1% of all the P-values) in salivary-gland than in other glands (n = 36 on chromosome 4), and we named them genes with salivary-specific expression. Then, we used a dynamic window approach to assess the density of salivary-specific expressed genes in every genomic region. Each window was selected to include exactly 15 genes. *MUC7* locates in the cluster with the largest number of genes with salivary-specific expression in chromosome 4 (Fig. 2A,B).

### PAML

To investigate deviations from neutrality in mammalian lineages, we conducted PAML^47^ using codeml program under branch site model A (allowing ω values to vary both among site and across branches), and compared with null model with fix ω = 1. Each branch in the phylogeny was tested separately. The difference of the -lnL values were compared by Chi-square test, and alpha-value = 0.05 (if P-value < 0.05 we reject the null hypothesis). The data can be found in Table S5.

## Additional Information

**How to cite this article**: Xu, D. *et al*. Recent evolution of the salivary mucin MUC7. *Sci. Rep.* **6**, 31791; doi: 10.1038/srep31791 (2016).

## Supplementary Material

Supplementary Information

Supplementary Table S1

Supplementary Table S2

Supplementary Table S3

Supplementary Table S4

Supplementary Table S5
