# Supplementary material for: Recent evolution of the salivary mucin MUC7
Source: Sci Rep. 2016 Aug 25;6:31791. doi: 10.1038/srep31791 (PMC4997351; doi:10.1038/srep31791)
Supplement: Supplementary Information [file srep31791-s1.pdf]

SUPPLEMENTARY INFORMATION

Recent evolution of the salivary mucin *MUC7*

Duo Xu[1], Pavlos Pavlidis[2], Supaporn Thamadilok[3], Emilie Redwood[1], Sara Fox[1], Ran Blekhman[4], Stefan Ruhl[3], Omer Gokcumen[1]**

Affiliations
[1]Department of Biological Sciences, State University of New York at Buffalo, New York 14260, USA
[2]Institute of Molecular Biology and Biotechnology (IMBB), Foundation of Research and Technology--Hellas, Heraklion, Crete, Greece
[3]Department of Oral Biology, School of Dental Medicine, State University of New York at Buffalo, New York 14214, USA
[4]Department of Genetics, Cell Biology, and Development, University of Minnesota, Twin Cities, Minnesota 55455, USA

Correspondence and requests for materials should be addressed to O.G. (email: omergokc@buffalo.edu) or to S.R. (email: shruhl@buffalo.edu)

Supplementary Figure 1. BLASTn results for the first coding exon (the second exon, which codes for the signal peptide) of human *MUC7*. Y-axis plots the -$\log_{10}$(E-value), X-axis shows the species with targeted sequence. Only placental mammals have sequences with E-value < 0.01. There are no hits (even with the lowest E-value threshold) to Marsupial genomes. For reference, we plotted them as 0 in a different color at the end of the plot. We also conducted a similar analysis for the *histatin*-like sequence. We found this sequence only in primates and obtained no hits in non-primate mammals or vertebrates (green dots indicate no hit).

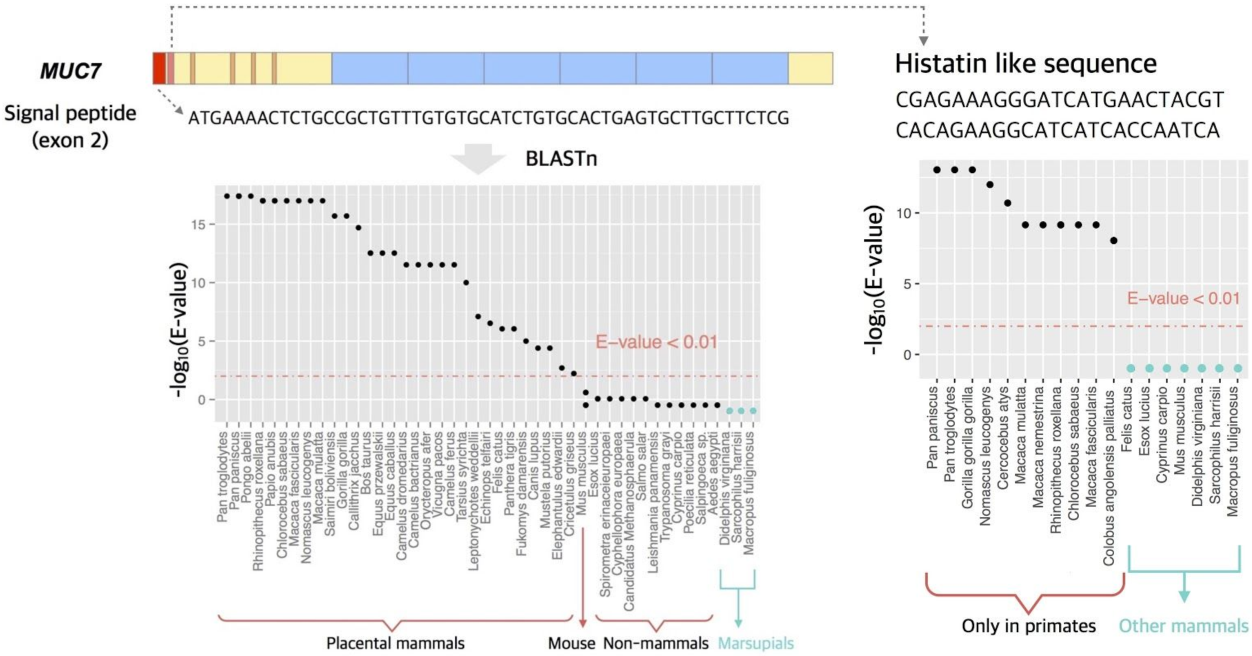

Supplementary Figure 2. MUC5B and MUC7 comparison of percentage of T and S residues when compared to all amino acids within the gene, which indicates the potential *O*-glycosylation sites. The figure shows this percentage for orthologs of *MUC7*, *MUC5B*, among primates and placental mammals. Statherin (STATH), which is encoded in the same chromosomal location and which is also a member of the SCPP family of proteins, but is not a mucin, was added as an comparison.

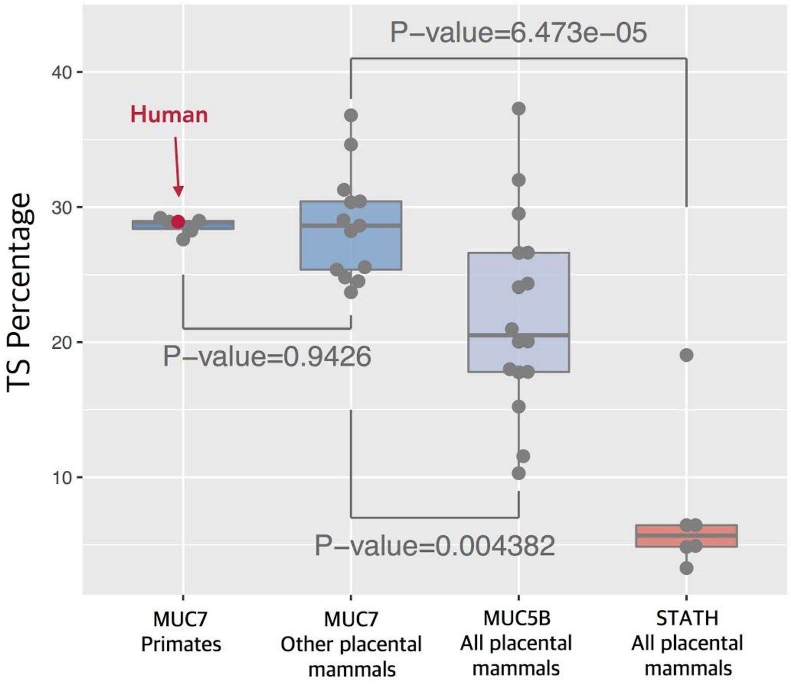

Supplementary Figure 3. Divergence of both copy number variation and repeat sequence among all proteins containing exonic tandem repeats. As a source for our analysis, we used data from Schaper et al. (2014), who performed a comparative analysis of 3,091 human subexonic tandem repeats. The authors categorized these repeats based on their divergence from orthologous repeats among eukaryotes for both sequence and copy number variation. Here, we constructed a "violin" plot based on their data. The thickness of the plot indicates the number of observations for that y-axis value. The y-axis shows the number of evolutionary steps (clades) it takes an exonic repeat sequence to show complete divergence as compared to humans. The divergence in *MUC7* copy number and PTS-repeat (TR) sequence are indicated by red dots. The subexonic tandem repeats of *MUC7* are in the highest 0.4% and 17% divergence among all subexonic repeats in the human genome for nucleotide and copy number variation, respectively as compared to rest of the eukaryota. For example, *MUC7* showed complete divergence for copy number when human *MUC7* is compared to other homininae species compared. In other words, subexonic repeats that show high divergence would be at the bottom, while highly conserved. As noted by Schaper et al. (2014), majority of subexonic repeats are highly conserved and divergene is observed only when human subexonic repeat sequences are compared with distant eukaryotic species (e.g. protozoa).

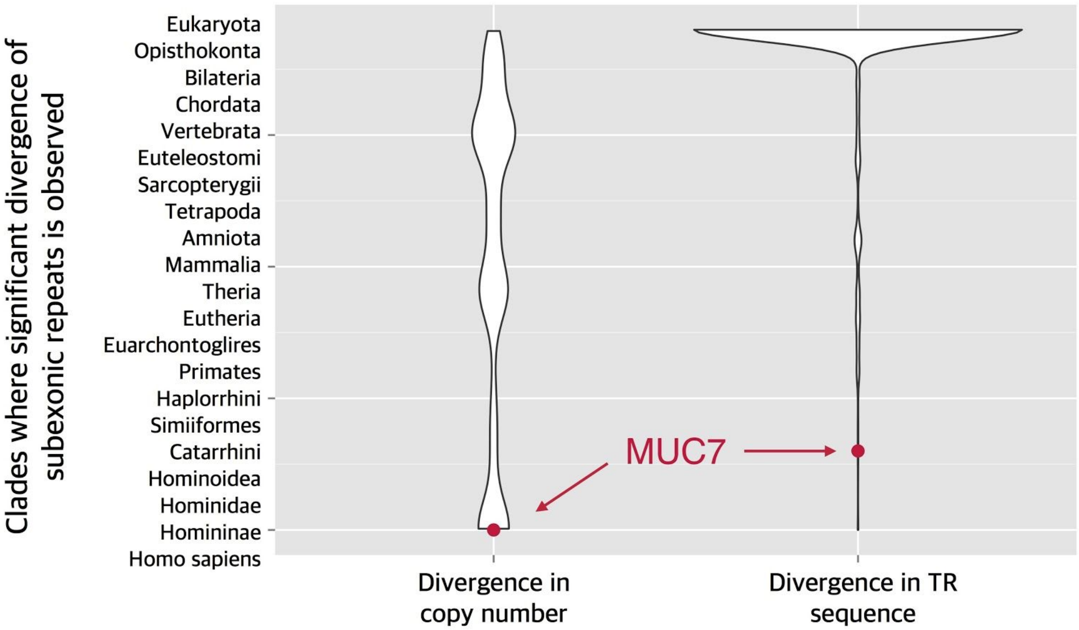

Supplementary Figure 4. Phylogenetic tree of the primate PTS repeats. We named the PTS repeats by the first two letters of the species name and order of appearance in the reference genome starting from the 5’-end (e.g., Ch-2 is the 2nd repeat from the 5’ end in the chimpanzee genome). Using this split data file, we constructed a phylogenetic tree with 100 bootstrap replicates. For instance, we observed that Or-2, Or-3, Or-4 and Or-5 are results of duplications in the orangutan lineage from an ancestral orangutan repeat orthologous to Hu-6, Ch-5 and Go-5. Similarly, Go-2 and Go-4 have evolved specifically in the gorilla lineage from an ancestral repeat orthologous to Hu-2 and Ch-2. In contrast, some lineage specific repeats, such as Hu-5 and Ch-4, which likely evolved in human-chimpanzee ancestors do not appear to have immediate orthologs in gorilla and orangutan genomes.

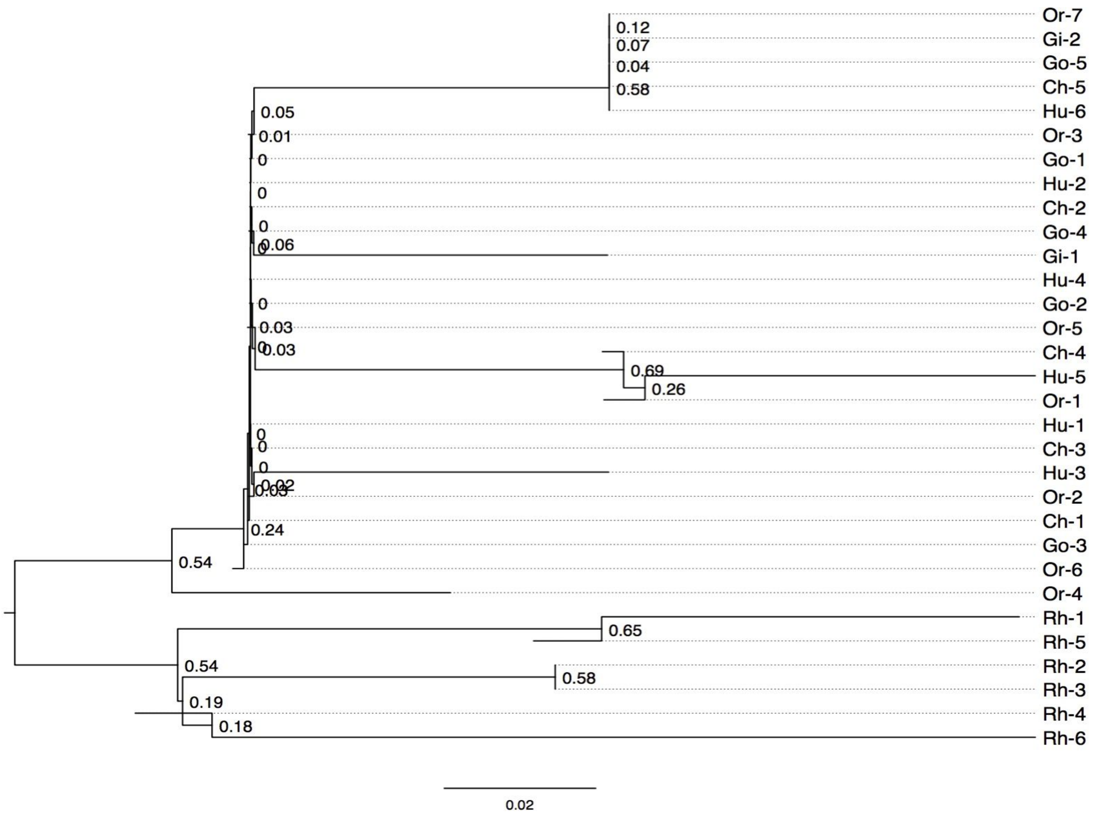

Supplementary Figure 5. Pairwise synonymous changes between each repeat within and among primate *MUC7* PTS-repeats. Different colors on the right and bottom axes indicate different species. The number shows the position of those repeats in their *MUC7* repetitive regions (e.g., Rh_1 indicates the first repeat from the 5' in the rhesus macaque reference genome). The colors in the heatmap show the synonymous changes between each repeat, with warmer colors indicating higher number of nucleotide differences between any two repeats. The groupings (clusters) shown on top and to the left of the heatmap were constructed based on sequence similarity without any *a priori* hypothesis.

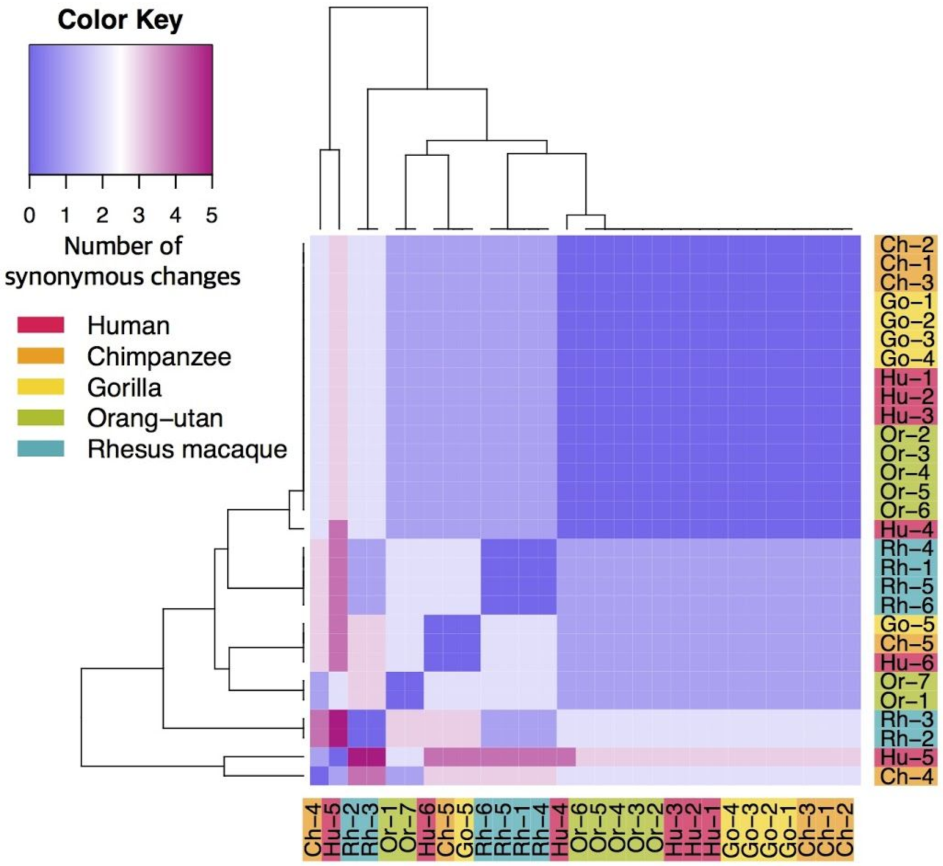

Supplementary Figure 6. The conservation of potential O-glycosylation sites in individual PTS-repeats in primates. We used published experimental data on GalNAc transferase activities on human MUC7 T and S amino acids[39] to evaluate the conservation of potential glycosylation sites in MUC7 PTS-repeats. The columns show the amino acids in each MUC7 PTS-repeat in each of the species. Activities of the GalNAc-transferase enzymes (-1, -2, -3, -4, -6), which were shown to glycosylate MUC7 repeat peptides in vitro at given sites are shown on top of each column[29]. Serines (S) and Threonines (T) are indicated by red and blue, respectively. Prolines (P) are highlighted in gray as they increase the rigidity of the peptide and were shown to contextually enhance adjacent GalNAc transferase activity and thus O-glycosylation.

blue and pink highlights show the O-glycosylation sites/units.

Amino acid position	1	2	3	4	5	6	7	8	9	10	11	12	13	14	15	16	17	18	19	20	21	22	23	24
GalNAc-T Activity	0.7	2.2					12.5			8.8		3.3	17.1					5.8	9.3	10.4				
Human-repeat-1	T	T	A	A	P	P	T	-	P	S	A	T	T	P	A	P	P	S	S	S	A	P	P	E
Human-repeat-2	T	T	A	A	P	P	T	-	P	S	A	T	T	Q	A	P	P	S	S	S	A	P	P	E
Human-repeat-3	T	T	A	A	P	P	T	-	P	P	A	T	T	P	A	P	P	S	S	S	A	P	P	E
Human-repeat-4	T	T	A	A	P	P	T	-	P	S	A	T	T	P	A	P	L	S	S	S	A	P	P	E
Human-repeat-5	T	T	A	V	P	P	T	-	P	S	A	T	T	L	D	P	S	S	A	S	A	P	P	E
Human-repeat-6	T	T	A	A	P	P	T	-	P	S	A	T	T	P	A	P	P	S	S	P	A	P	Q	E
Chimpanzee-repeat-1	T	T	A	A	P	P	T	-	P	S	A	T	T	P	A	P	P	S	S	S	A	P	P	E
Chimpanzee-repeat-2	T	T	A	A	P	P	T	-	P	S	A	T	T	Q	A	P	P	S	S	S	A	P	P	E
Chimpanzee-repeat-3	T	T	A	A	P	P	T	-	P	S	A	T	T	P	A	P	P	S	S	S	A	P	P	E
Chimpanzee-repeat-4	T	T	A	V	P	P	T	-	P	S	A	T	T	L	D	P	P	S	A	S	A	P	P	E
Chimpanzee-repeat-5	T	T	A	A	P	P	T	-	P	S	A	T	T	P	A	P	P	S	S	P	A	P	Q	E
Gorilla-repeat-1	T	T	A	A	P	P	T	-	P	S	A	T	T	P	A	P	P	S	S	S	A	P	P	E
Gorilla-repeat-2	T	T	A	A	P	P	T	-	P	S	A	T	T	Q	A	P	P	S	S	S	A	P	P	E
Gorilla-repeat-3	T	T	A	A	P	P	T	-	P	S	A	T	T	P	A	P	P	S	S	S	A	P	P	E
Gorilla-repeat-4	T	T	A	A	P	P	T	-	P	S	A	T	T	Q	A	P	P	S	S	S	V	P	P	E
Gorilla-repeat-5	T	T	A	V	P	P	T	-	P	S	A	T	T	P	A	P	P	S	S	P	A	P	Q	E
Orangutan-repeat-1	T	T	A	A	P	P	T	-	P	S	A	T	T	P	A	P	P	S	A	S	A	P	P	E
Orangutan-repeat-2	T	T	A	A	P	P	T	-	P	S	A	T	T	P	A	P	P	S	S	S	A	P	Q	D
Orangutan-repeat-3	T	T	A	A	P	P	T	-	P	S	A	T	T	P	A	P	P	S	S	S	A	P	Q	D
Orangutan-repeat-4	T	T	A	A	P	P	T	-	P	S	A	T	T	T	A	P	P	S	S	S	A	P	Q	D
Orangutan-repeat-5	T	T	A	A	P	P	T	-	P	S	A	T	T	P	A	P	P	S	S	S	A	P	P	D
Orangutan-repeat-6	T	T	A	A	P	P	T	-	P	S	A	T	T	P	A	P	P	S	S	S	A	P	P	E
Orangutan-repeat-7	T	T	A	A	P	P	I	-	P	S	A	T	T	P	A	P	P	S	S	P	A	P	Q	E
Rhesus-repeat-1	T	T	A	P	P	P	T	-	P	S	A	T	T	A	A	L	P	P	S	S	A	P	P	E
Rhesus-repeat-2	T	T	T	A	P	P	I	-	P	S	A	T	T	P	A	P	P	P	S	S	A	P	P	E
Rhesus-repeat-3	T	T	T	A	P	P	T	-	P	S	A	T	T	P	A	P	P	P	S	S	A	P	P	E
Rhesus-repeat-4	T	T	A	A	P	P	T	-	P	S	A	T	T	P	A	L	P	P	S	S	A	P	P	E
Rhesus-repeat-5	T	T	A	A	P	P	T	-	P	S	A	T	T	A	V	L	P	P	S	S	A	P	P	E
Rhesus-repeat-6	T	T	A	A	P	I	T	T	P	S	A	T	T	P	A	P	P	P	S	S	A	P	V	E

Supplementary Figure 7. Variation of copy number range among humans, chimpanzees, gorillas and orangutan genomes. The boxplot is the result of 1,000 simulations given the phylogenetic tree and the mutation rate (single loss or gain in a million years). The red dots indicate all observed variations.

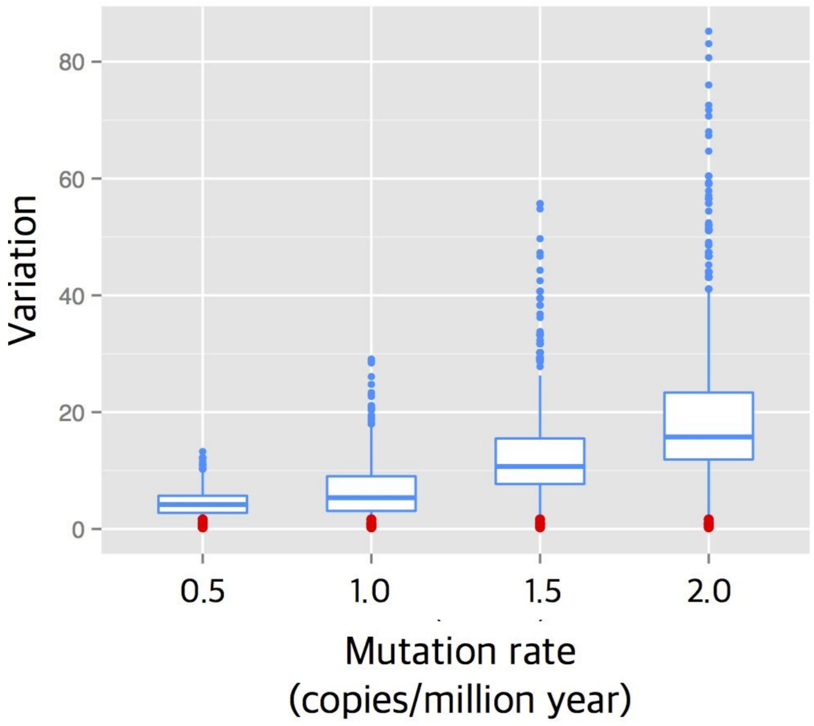

Supplementary Figure 8. Phylogeny of *MUC7* gene without repeat region (Maximum likelihood tree, with 500 bootstrap replicates). Here, we used the reference genome alignments of the *MUC7* coding regions outside of the PTS-repeat region to build this tree. Note the complete separation of primates from other mammalian species. The red text indicate the P-value from PAML test rejecting neutrality (detailed explanations can be found in Methods and Table S5), indicating positive selection. In other branches, we did not observe any significant indication of positive selection.

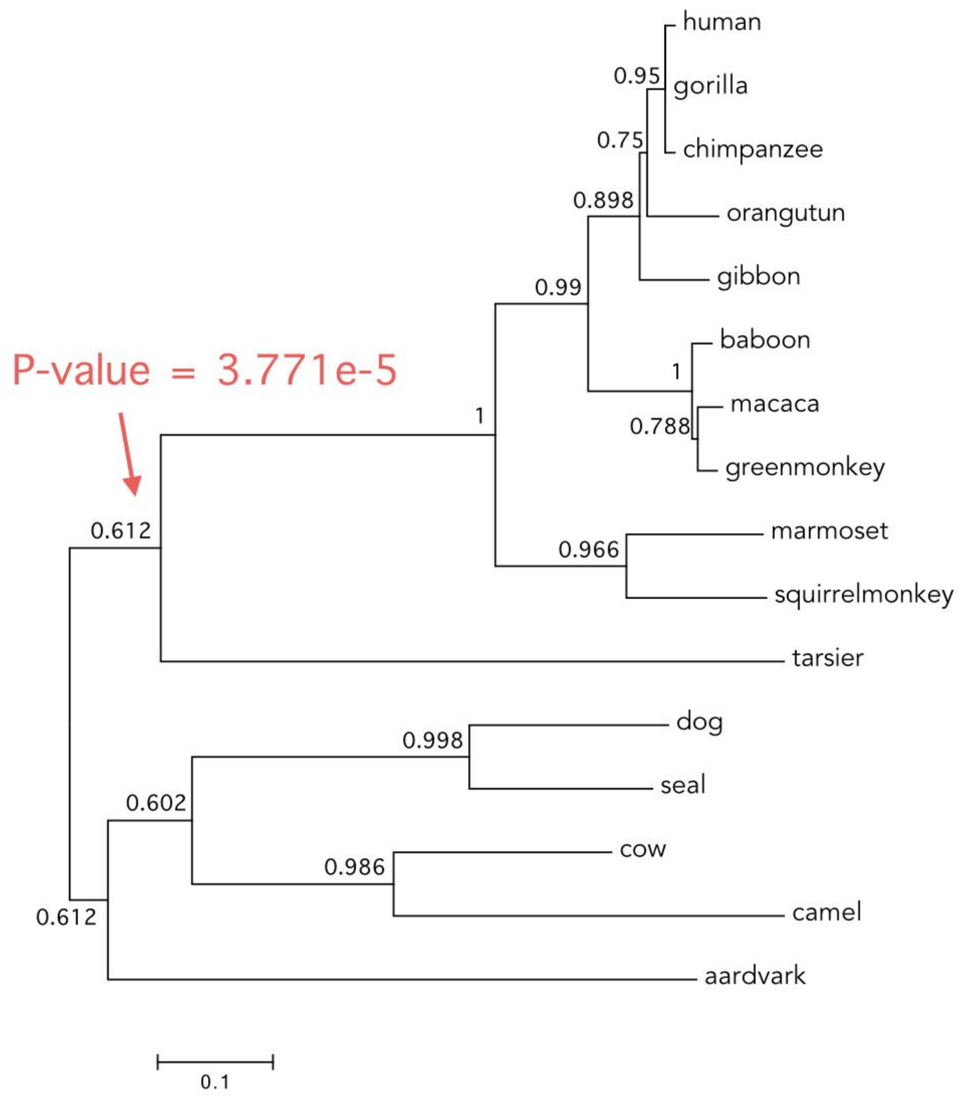

Supplementary Tables
These are uploaded separately

Supplementary Table 1. Gene cluster with significant high salivary-specific expression

Supplementary Table 2. Repeat number and TS count for mammals and primers used for the study.

Supplementary Table 3. Non-human primates copy number states genotyped by PCR.

Supplementary Table 4. Simulation of copy number for great apes under different copy number gain-loss rate.

Supplementary Table 5. Results for PAML analysis.
